# Navigating the Post-opioid Era: A Focus on Non-opioid Perioperative Analgesics

**DOI:** 10.7759/cureus.101209

**Published:** 2026-01-10

**Authors:** Ahmed A Al-Awadhi, Saraa Yoosuf, Iana A Malasevskaia

**Affiliations:** 1 Anaesthesiology, German-Yemeni Hospital, Sana'a, YEM; 2 Harvard Medical School, Harvard University, Boston, USA; 3 Principles and Practice of Clinical Research (PPCR), Harvard T.H. Chan School of Public Health, Boston, USA

**Keywords:** glucocorticoids, ketamine, lidocaine, multimodal analgesia, non-opioid analgesia, nsaids, opioid-sparing, perioperative pain, postoperative recovery, regional anesthesia

## Abstract

The substantial adverse effects associated with opioid-based analgesia and its contribution to postoperative dependence have prompted a shift toward multimodal, opioid-sparing perioperative strategies. Non-opioid analgesics now form the cornerstone of contemporary perioperative management and Enhanced Recovery After Surgery (ERAS) pathways. This review synthesizes current evidence on the efficacy, safety, and clinical utility of major non-opioid analgesic classes, including nonsteroidal anti-inflammatory drugs (NSAIDs), acetaminophen, N-methyl-D-aspartate (NMDA) antagonists, intravenous lidocaine, gabapentinoids, α2-agonists, regional anesthesia techniques, and glucocorticoids, for postoperative pain management. Evidence from randomized controlled trials, systematic reviews, and meta-analyses was evaluated with emphasis on analgesic effectiveness, opioid-sparing capacity, recovery outcomes, and adverse effects.

The efficacy of non-opioid multimodal analgesia (MMA) stems from the synergistic targeting of distinct pain pathways. Across drug classes, non-opioid agents demonstrate clinically meaningful opioid-sparing effects while providing analgesia that is comparable to opioid-based regimens. Acetaminophen and NSAIDs are cornerstones of MMA, supported by extensive high-quality evidence. NMDA antagonists such as ketamine show particular benefit in major surgical procedures for modulating central sensitization and preventing chronic pain, whereas intravenous lidocaine has unique advantages in accelerating gastrointestinal recovery and may reduce hospital length of stay. Gabapentinoids may serve as adjuncts but exhibit heterogeneous efficacy and a side-effect profile (e.g., sedation) that necessitates selective use. Preoperative α2-agonists consistently prolong analgesia and reduce perioperative opioid requirements. As a core component of MMA, regional anesthesia techniques demonstrate robust reductions in both prolonged postoperative opioid use and the incidence of chronic postsurgical pain, and perioperative glucocorticoids such as dexamethasone contribute potent dual analgesic and antiemetic effects.

The collective evidence indicates that MMA, integrating agents with complementary mechanisms, provides superior pain control, enhanced functional recovery, and meaningful reductions in opioid exposure. Broader implementation of standardized, procedure-specific multimodal protocols may further decrease opioid-related harms and strengthen alignment with ERAS principles. Future research should prioritize long-term outcomes and optimization of multimodal combinations to advance the transition toward a post-opioid paradigm in surgical care.

## Introduction and background

Postoperative pain remains one of the most common and challenging complications in the perioperative period. Although prevalence varies by surgery type, systematic reviews suggest a high global burden, with estimates indicating that up to 80% of surgical patients experience acute pain and more than half report moderate to severe intensity [1]. With over 300 million surgeries performed each year globally, the effective management of this acute pain is a critical healthcare priority [2]. Inadequate postoperative analgesia is consistently associated in the literature with numerous adverse outcomes, including delayed recovery, impaired wound healing, increased cardiopulmonary complications, prolonged hospitalization, and, with evidence supporting a potential causal role, the development of persistent postsurgical pain (PPSP) [1].

In the United States, opioid-based analgesia has traditionally been the foundation of postoperative pain management. However, the significant risks of this approach, including respiratory depression, sedation, nausea, and constipation, are now widely recognized. More critically, postsurgical opioid prescribing has been identified as a major contributor to the modern opioid epidemic [2]. This US-centric practice pattern stands in contrast to global trends, yet the evidence for opioid-sparing strategies is derived from international research and is broadly applicable. The disproportionately high postoperative opioid prescribing in US settings underscores an urgent need for a paradigm shift toward multimodal, opioid-sparing strategies, consistent with global best practices [2].

This shift is embodied by the principles of Enhanced Recovery After Surgery (ERAS) protocols, which aim to optimize patient outcomes through evidence-based, multimodal care pathways [3]. As outlined in ERAS guidelines from the ERAS® Society, a fundamental principle is multimodal opioid-sparing analgesia [3]. This strategy utilizes a combination of pharmacological agents and regional anesthetic techniques to modulate nociception through synergistic physiological pathways [3]. This methodology optimizes pain control while significantly reducing opioid consumption and the consequent risk of related adverse effects. Standard non-opioid adjuncts encompass nonsteroidal anti-inflammatory drugs (NSAIDs), acetaminophen, gabapentinoids, glucocorticoids, and ketamine. Tramadol, a weak opioid with additional monoaminergic activity, may also be incorporated into multimodal regimens but is not considered a first-line non-opioid agent. These pharmacological adjuncts are frequently integrated with regional techniques, including neuraxial analgesia and peripheral nerve blocks, to provide comprehensive perioperative analgesia [3].

**Figure 1:**
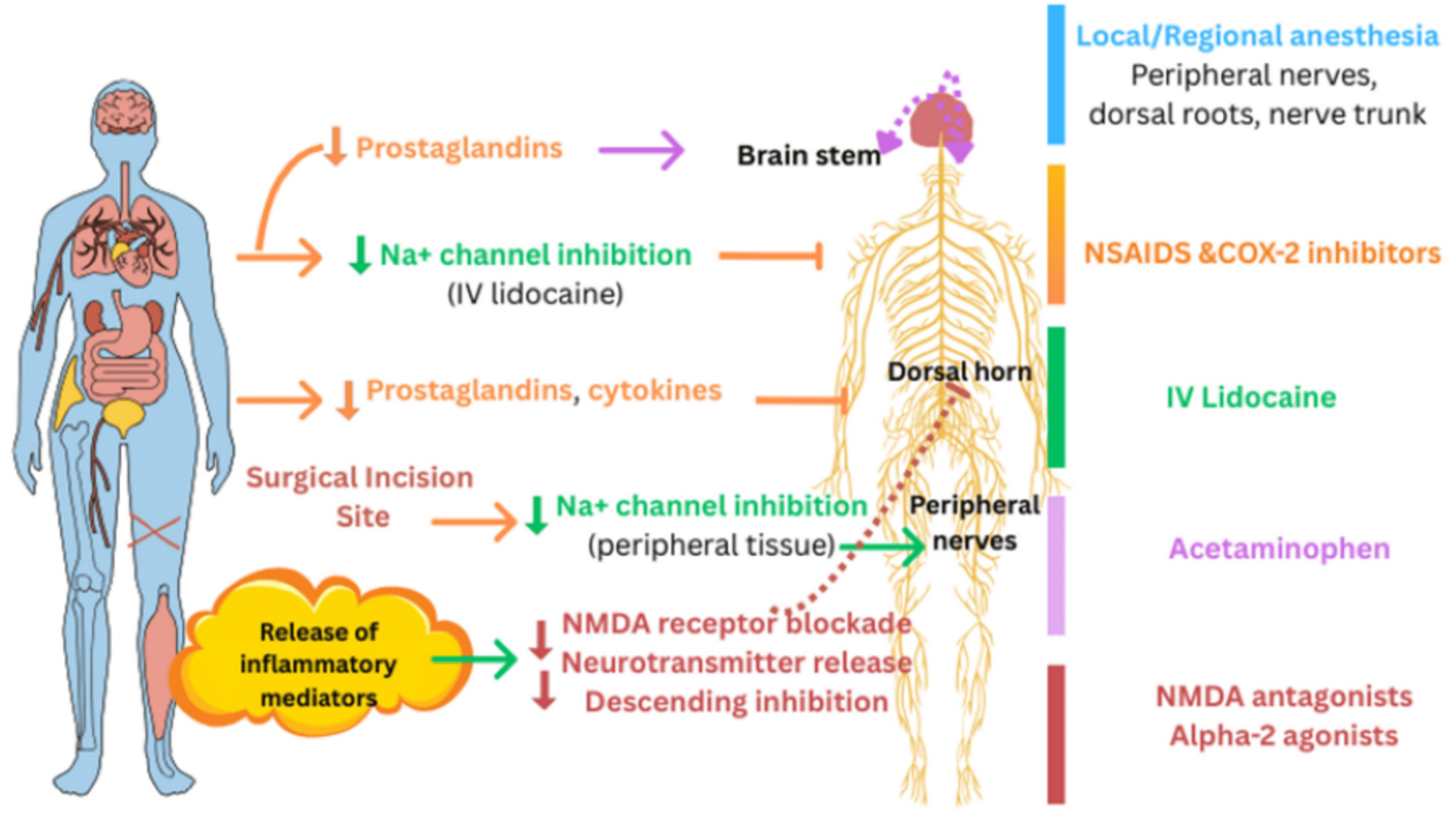
Mechanistic Framework of Multimodal Perioperative Analgesia Color coding indicates the primary mechanism of action: blue denotes anatomical neural conduction blockade (local/regional anesthesia); green represents pharmacologic ion channel modulation (IV lidocaine); orange indicates modulation of inflammatory pathways (NSAIDs, cytokines); purple reflects central analgesic modulation (acetaminophen); and red denotes spinal and central synaptic modulation (NMDA antagonists and α2-agonists). COX = cyclooxygenase; NSAIDs = nonsteroidal anti-inflammatory drugs; NMDA = N-methyl-D-aspartate; IV = intravenous; Na⁺ = sodium; Ca²⁺ = calcium. This illustration was created using Canva by Iana A. Malasevskaia.

A growing body of evidence supports the effectiveness of these strategies [4]. Numerous pharmacological classes, such as local anesthetics (e.g., lidocaine), NSAIDs and COX-2 inhibitors (e.g., ketorolac, celecoxib), α2-agonists (clonidine, dexmedetomidine), and NMDA antagonists (ketamine, magnesium), have demonstrated significant analgesic and opioid-sparing benefits in a variety of surgical contexts, although the magnitude of benefit can vary across specific procedures and study designs (Figure 1). These strategies align with contemporary recommendations, such as those from the Centers for Disease Control and Prevention (CDC), which prioritize non-opioid therapies to mitigate the harms associated with opioid overuse [4].

To contextualize the mechanistic rationale for multimodal perioperative analgesia, Figure 1 illustrates the primary nociceptive pathways activated during surgical injury and the specific sites at which key non-opioid pharmacologic agents and regional anesthesia exert their effects.

Despite this expanding adoption and supporting evidence, the available literature remains heterogeneous. Studies vary widely in key methodological aspects, including dosing regimens (e.g., timing, duration, and dose), surgical procedures (e.g., major abdominal vs. outpatient orthopedic procedures), and measured outcomes (e.g., pain scores, opioid consumption, and functional recovery). This variability leads to inconsistent findings and complicates clinical interpretation, limiting the ability to determine which non-opioid modalities offer the most reliable balance of efficacy and safety.

Therefore, a comprehensive synthesis of the current evidence is essential. This review aims to synthesize and evaluate the literature on the efficacy and safety of non-opioid perioperative analgesics across diverse surgical populations, with a specific focus on their capacity to provide effective pain relief, reduce opioid requirements, and enhance overall recovery outcomes. A primary emphasis is placed on evaluating safety tradeoffs and the risk-benefit profile of each pharmacological class within multimodal regimens.

## Review

This review was conducted as a narrative synthesis of the current evidence on non-opioid perioperative analgesia. A literature search was performed using PubMed, MEDLINE, and Google Scholar from database inception to December 5, 2025. The search was restricted to articles published in English. Search terms included “non-opioid analgesia,” “multimodal analgesia,” “postoperative pain management,” “opioid-sparing,” and specific drug-class terms (e.g., “ketamine,” “lidocaine infusion,” “NSAIDs,” “gabapentinoids,” “α2-agonists”). Eligible studies included randomized controlled trials (RCTs), systematic reviews, meta-analyses, and high-quality observational studies reporting postoperative analgesic efficacy, opioid-sparing outcomes, recovery metrics, or adverse events. Studies were screened for relevance to perioperative pain management and multimodal strategies; pediatric-only, chronic pain, and non-surgical studies were excluded. In synthesizing the evidence, greater weight was given to findings from RCTs, systematic reviews, and meta-analyses, while observational studies were used to support or contextualize these findings. Data were extracted narratively and organized by analgesic class to facilitate comparative interpretation of mechanisms, clinical effects, and safety profiles.

The efficacy of non-opioid perioperative analgesia is rooted in the synergistic use of medications targeting distinct pain pathways. This section categorizes the evidence by major drug class, summarizing the mechanism of action and synthesizing the key findings on their analgesic efficacy, opioid-sparing effects, and role in recovery.

Foundational Agents: NSAIDs and Acetaminophen

NSAIDs are a cornerstone of multimodal perioperative analgesia, functioning primarily through the inhibition of cyclooxygenase (COX) enzymes and thereby reducing the synthesis of prostaglandins that mediate pain, inflammation, and fever [5]. The evidence strongly supports their analgesic efficacy and opioid-sparing role. A meta-analysis by De Oliveira et al. (2012) established that a single perioperative dose of ketorolac, particularly the 60 mg formulation, significantly decreases early postoperative pain and opioid consumption [6]. While the 60 mg dose showed significant benefit in this analysis, the authors note the importance of dose selection based on individual patient risk factors, given the known dose-dependent risks associated with NSAIDs [6]. Early clinical trials by Kenny et al. demonstrated that in patients undergoing upper abdominal surgery, ketorolac provided analgesia equivalent to opioids such as alfentanil while avoiding associated cardiorespiratory depression and significantly reducing postoperative patient-controlled analgesia (PCA) morphine requirements [7]. These findings highlight the efficacy of NSAIDs in a major surgical context, though the magnitude of opioid-sparing effects may vary across different surgical types.

This foundational evidence is reinforced by modern trials across diverse surgical specialties. For instance, Papoian et al. demonstrated that a regimen of acetaminophen and ibuprofen after thyroidectomy provided pain control equivalent to an opioid-based regimen while markedly lowering morphine use [8]. Similarly, a non-inferiority RCT by DeMasy et al. found that a ketorolac-centered non-opioid protocol was not only non-inferior but also resulted in significantly lower average pain scores compared to opioid therapy after nephrolithiasis surgery [9]. The utility of NSAIDs extends to major operations as well. As shown by Lukonina et al., diclofenac and ibuprofen served as effective components of non-opioid regimens in complex spine surgery, leading to lower pain scores, fewer breakthrough opioid doses, and shorter durations of opioid therapy [10]. Despite their well-established benefits, the known risks associated with COX-1 inhibition, including gastrointestinal toxicity and platelet dysfunction, necessitate careful patient selection [5].

Acetaminophen is a central non-opioid analgesic and antipyretic with a mechanism distinct from traditional NSAIDs. Although not fully defined, it likely involves central COX inhibition, activation of descending serotonergic pathways, and modulation of the endocannabinoid system, producing analgesic and antipyretic effects without meaningful peripheral anti-inflammatory activity [11]. Its contribution within multimodal perioperative regimens is well supported. The RCT by Papoian et al. demonstrated that postoperative acetaminophen and ibuprofen provided analgesia equivalent to an opioid-based regimen after thyroidectomy [8]. Furthermore, the study by Beloeil et al. highlighted its synergistic potential: when combined with nefopam and ketoprofen (the PNK regimen), acetaminophen contributed to a substantial reduction in 24-hour morphine consumption (5 mg vs. 27 mg with placebo) and produced superior analgesia [12]. These findings establish acetaminophen as a fundamental and effective component of opioid-sparing strategies, valued for its excellent safety profile when used within recommended dosing limits [11].

Adjuvants for Major Surgery and Central Sensitization

In adult surgical patients, NMDA receptor antagonists, such as ketamine and magnesium, play a critical role in modulating central sensitization and preventing pain chronification [13]. Antagonism of these receptors can prevent and reverse neuronal hyperexcitability (“wind-up”), providing analgesia that is particularly effective in opioid-tolerant states and neuropathic pain [13]. The evidence supports their significant opioid-sparing and analgesic efficacy in major surgery. The RCT by Feld et al., which employed a complex non-opioid regimen including ketamine and magnesium sulfate for gastric bypass surgery, resulted in significantly reduced postoperative sedation, lower PACU morphine consumption, and higher patient satisfaction compared to a fentanyl-based regimen [14]. These findings demonstrate that NMDA antagonism can effectively replace a substantial portion of intraoperative opioid requirements.

The synergistic value of this drug class is further highlighted by Beloeil et al., in which nefopam, a non-opioid analgesic with recognized NMDA-modulating properties, contributed to a profound morphine-sparing effect when combined with paracetamol and ketoprofen [12]. Additional support comes from the study by Tumanyan et al., whose preemptive multimodal regimen that included magnesium sulfate and nefopam achieved a remarkable 96% opioid-sparing effect in gynecologic oncology surgery [15]. Among NMDA antagonists, ketamine remains the agent with the most robust and consistent perioperative evidence, while the data for magnesium are more limited, heterogeneous, and less consistent. Application of this class extends beyond adults; a systematic review by Persad et al. evaluated ketamine for procedural pain in neonates and found a significant reduction in pain scores compared to placebo, although the “very low-certainty” of evidence underscores the need for further pediatric research [16].

Intravenous Lidocaine

Intravenous (IV) lidocaine represents a unique analgesic strategy, repurposing a sodium channel blocker for systemic analgesic, anti-hyperalgesic, and anti-inflammatory effects. Its primary mechanism involves the blockade of voltage-gated sodium channels, but it also exhibits multimodal actions, including NMDA receptor modulation and attenuation of inflammatory pathways [17]. The strongest evidence derives from large-scale systematic reviews. Weibel et al. and Kranke et al. collectively analyzed data from thousands of patients and consistently found that IV lidocaine reduces early postoperative pain, provides a significant opioid-sparing effect, and, most notably, accelerates gastrointestinal recovery by reducing the time to first flatus and first bowel movement [18,19]. While systematic reviews note that IV lidocaine does not demonstrate a clear advantage over epidural analgesia in some major abdominal surgeries, its ease of administration and avoidance of neuraxial technique-related complications support its integration into ERAS pathways, particularly where epidural analgesia is contraindicated or impractical [17].

This pro-kinetic effect makes IV lidocaine especially valuable within ERAS protocols for abdominal surgery [17]. Its clinical feasibility is demonstrated by its incorporation into the non-opioid regimen used by Feld et al. in gastric bypass surgery [14] and in the preemptive multimodal protocol evaluated by Tumanyan et al. in gynecologic oncology surgery [15]. Due to its relatively narrow therapeutic index, IV lidocaine requires careful patient selection, with key contraindications including severe cardiac conduction disorders, hepatic impairment, and seizure disorders, and continuous monitoring of neurological status, electrocardiogram (ECG), and blood pressure to ensure safety [17].

Specialized Adjuncts: Gabapentinoids and Alpha-2 Agonists

Gabapentinoids (gabapentin and pregabalin) bind the α2δ-1 subunit of voltage-gated calcium channels, thereby reducing excitatory neurotransmitter release and attenuating central sensitization [20]. Their role in acute postoperative analgesia is context-dependent and appears most effective when incorporated into a broader multimodal regimen. A retrospective study by Nguyen et al. found substantial benefit when gabapentin was paired with celecoxib in aesthetic plastic surgery, significantly reducing rescue meperidine requirements and the need for antiemetics [21]. However, an RCT by Long-Lijoi et al. offers an important contrast. While gabapentin-based and NSAID-based regimens provided equivalent analgesia in cosmetic surgery, the NSAID protocol produced higher patient satisfaction, better compliance, fewer bleeding-related complications, and a shorter PACU stay [22]. These findings indicate that although gabapentinoids are effective analgesic adjuncts, they may not be optimal in settings where rapid recovery is essential, particularly given their side-effect profile (e.g., sedation, dizziness). This underscores the importance of patient selection, suggesting that gabapentinoids should be avoided or used cautiously in patients for whom sedation or delayed emergence may compromise recovery goals, such as in fast-track or outpatient surgical settings.

Alpha-2 adrenergic agonists (clonidine and dexmedetomidine) provide analgesia primarily through activation of central presynaptic α2 receptors, resulting in reduced norepinephrine release and attenuation of pain signal transmission [23]. A defining feature of this class is its significant opioid-sparing effect. This is strongly supported by a meta-analysis by Ju et al., which found that a single preoperative systemic dose significantly reduced postoperative opioid consumption and pain intensity for up to 24 hours [24]. This sustained benefit suggests a role in preemptive analgesia and perioperative anxiolysis. These findings are consistent with Feld et al., who incorporated a substantial IV dose of clonidine as a key component of their opioid-free regimen for gastric bypass surgery [14]. While α2-agonists offer multiple advantages, their use requires careful titration and monitoring for potential bradycardia and hypotension [23]. Optimal dosing should be individualized, as hemodynamic responses may vary based on patient factors and concurrent medications.

Regional Anesthesia Techniques

Regional anesthesia emerges as a promising strategy in the post-opioid era, offering targeted pain control while reducing reliance on systemic opioids. This approach is supported by systematic evidence demonstrating meaningful long-term benefits. A 2024 meta-analysis by Pepper et al. found that regional anesthesia significantly reduces prolonged opioid use (relative risk 0.48) and decreases chronic postsurgical pain for up to six months following surgery [25]. Complementing these long-term findings, a comprehensive review by Gupta et al. (2021) highlights the immediate clinical utility of specific regional techniques, such as erector spinae, pectoral nerves (PECS) II, and serratus anterior blocks, as effective opioid-sparing components across breast, cardiac, thoracic, and orthopedic surgeries [26].

Despite robust evidence supporting their efficacy, broader implementation of these techniques faces challenges, including the requirement for specialized training, ultrasound proficiency, and appropriate equipment. These barriers can limit adoption, particularly in resource-constrained or low-volume surgical centers. Nonetheless, when available, regional anesthesia represents a highly effective strategy for perioperative opioid minimization.

Glucocorticoids

Perioperative glucocorticoids, particularly dexamethasone, have emerged as versatile and potent components of multimodal analgesia (MMA), offering significant analgesic and opioid-sparing benefits in addition to their well-known antiemetic effects. Systematic reviews and meta-analyses provide strong and consistent evidence for their efficacy. An early meta-analysis by de Oliveira et al. (2011) demonstrated that dexamethasone reduces postoperative pain at rest and with movement within the first 24 hours and decreases opioid consumption, with intermediate (0.11-0.2 mg/kg) and high doses (≥0.21 mg/kg) producing the most reliable opioid-sparing effects [27].

These findings have been reinforced and expanded by more recent evidence. A 2024 meta-analysis by Laconi et al. (Table 1) confirmed that higher-dose dexamethasone regimens (≥0.2 mg/kg or ≥15 mg) significantly reduce 24-hour pain scores at rest and with movement, lower opioid requirements by approximately 10 mg of morphine equivalents, and markedly reduce postoperative nausea and vomiting (PONV) without increasing major adverse events [28].

**Table 1:** Summary of Key Studies on Non-opioid Perioperative Analgesics α2-agonists = alpha-2 adrenergic agonists; CI = confidence interval; ESB = erector spinae block; GI = gastrointestinal; IM = intramuscular; IPACK = infiltration between the popliteal artery and capsule of the knee; IV = intravenous; MD = mean difference; MgSO₄ = magnesium sulfate; MMA = multimodal analgesia; NR = not reported; NS = not significant; NSAIDs = non-steroidal anti-inflammatory drugs; OR = odds ratio; PACU = post-anesthesia care unit; PCA = patient-controlled analgesia; PECS = pectoral nerves; PNK = paracetamol-nefopam-ketoprofen; POD = postoperative day; PONV = postoperative nausea and vomiting; Preop = preoperative; RCT = randomized controlled trial; RR = risk ratio; SMD = standardized mean difference; SNRI = serotonin-norepinephrine reuptake inhibitor; TAP = transversus abdominis plane; TRPV1 = transient receptor potential vanilloid 1; WMD = weighted mean difference.

Author/Year	Design	Population (n)	Intervention(s)	Key Findings
Beloeil et al., 2019 [12]	RCT	Adults, major surgery (n=237)	Paracetamol, nefopam, ketoprofen (alone or combinations) vs placebo	PNK combination markedly reduced 24-h morphine use (5 mg vs 27 mg). Superior analgesia without ↑ adverse events.
DeMasy et al., 2022 [9]	Non-inferiority RCT	Adults, nephrolithiasis surgery (n=90)	Ketorolac vs oxycodone–acetaminophen	Non-opioid regimen non-inferior; lower pain scores (3.20 vs 4.17). No differences in constipation or leftover pills.
Feld et al., 2002 [14]	RCT	Obese adults, gastric bypass (n=30)	Multimodal non-opioid (ketorolac, clonidine, lidocaine, ketamine, MgSO₄) vs fentanyl	Lower PACU morphine (5.2 vs 7.8 mg/h) and less sedation. Higher satisfaction. No pain score difference.
De Oliveira et al., 2012 [6]	Systematic review and meta-analysis	Adults, various surgeries (n=782)	Single-dose ketorolac vs placebo	Lower pain (WMD –0.64), reduced opioid use, reduced PONV (OR 0.49). Stronger effect with 60 mg dose.
Ju et al., 2020 [24]	Systematic review and meta-analysis	Adults, non-cardiac surgery (n=748)	Preop α2-agonists (clonidine/dexmedetomidine) vs placebo	Reduced opioid use at 6 h (SMD –0.52) and 24 h (SMD –0.68); lower pain at 6 h and 24 h.
Kenny et al., 1990 [7]	Clinical trials (2)	Adults, minor and abdominal surgery (n=80)	Ketorolac (bolus or infusion) vs alfentanil or placebo	Analgesia comparable to opioids; significantly reduced PCA morphine; no cardiorespiratory depression.
Kranke et al., 2015 [19]	Systematic review	Adults, various surgeries (n=2802)	IV lidocaine infusion vs placebo/epidural	Reduced early pain (MD –0.84), faster GI recovery, lower opioid use and PONV. No advantage vs epidural.
Long-Lijoi et al., 2023 [22]	RCT	Adults, cosmetic surgery (n=106)	Gabapentin-based vs NSAID-based regimen	Equivalent analgesia. NSAID regimen had higher satisfaction, better compliance, fewer bleeding events, shorter PACU stay.
Lukonina et al., 2024 [10]	RCT	Adults, spine surgery (n=52)	Diclofenac–orphenadrine or ibuprofen vs tramadol	Lower pain on POD2, fewer breakthrough opioid doses, shorter opioid duration. High satisfaction; reduced cortisol (ibuprofen group).
Nguyen et al., 2018 [21]	Retrospective study	Adults, plastic surgery (n=462)	Celecoxib + gabapentin regimen vs opioids	Lower rescue opioid need (14.9% vs 44.6%), less antiemetic use, shorter PACU stay.
Papoian et al., 2020 [8]	RCT	Adults, thyroidectomy (n=95)	Acetaminophen + ibuprofen vs opioid-based care	Similar pain scores; opioid use significantly reduced (0.8 mg vs 6.9 mg).
Persad et al., 2023 [16]	Systematic review	Neonates (n=269)	Ketamine vs placebo/fentanyl	Ketamine reduced pain vs placebo; uncertain safety; very low-certainty evidence overall.
Tumanyan et al., 2019 [15]	RCT	Adults, gynecologic oncology surgery (n=97)	Preemptive lidocaine+MgSO₄+nefopam+NSAIDs vs opioid+NSAIDs	Opioid-sparing effect 96%; better pain control; fewer metabolic stress responses.
Weibel et al., 2018 [18]	Systematic review	Adults, various surgeries (n=4525)	IV lidocaine infusion	Reduced pain at all time points, ↓ opioid use, ↓ ileus risk, ↑ bowel recovery. No clear benefit vs epidural.
Pepper et al., 2024 [25]	Systematic review and meta-analysis	Adults undergoing noncardiac elective surgery; n = 348 for prolonged opioid use, n = 1489 and n = 3457 for chronic pain at different time points	Regional anesthesia for adults undergoing noncardiac elective surgery	Prolonged opioid use: RR 0.48, 95% CI 0.24-0.96, P = 0.04. Chronic postsurgical pain at 3 months: RR 0.74, 95% CI 0.59-0.93, P = 0.01. Chronic postsurgical pain at 6 months: RR 0.72, 95% CI 0.61-0.85, P < 0.001. No effect at 12 months: RR 0.44, 95% CI 0.16-1.17, P = 0.10
Gupta et al., 2021 [26]	Narrative review	Adults, various surgeries (evidence from multiple RCTs, meta-analyses, and trials)	Synthesis of emerging therapeutic options: Regional anesthesia (ESB, PECS II, Serratus, IPACK), non-opioid adjuncts (IV lidocaine, esmolol, SNRIs), novel opioids (oliceridine, tapentadol), novel formulations, non-pharmacologic therapy.	Regional anesthesia: Effective opioid-sparing across breast, cardiac, thoracic, and orthopedic surgeries. Non-opioid adjuncts: IV lidocaine reduces early pain/opioid use and accelerates GI recovery; esmolol reduces intra/postoperative opioid use; duloxetine reduces opioid use up to 48 h. Novel opioids: Oliceridine reduces PONV vs. morphine; tapentadol provides analgesia with fewer GI side effects. Non-pharmacologic: Relaxation, acupuncture, TENS reduce pain and opioid need. The review concludes multimodal, opioid-sparing strategies are essential for improving recovery and mitigating opioid-related harms.
de Oliveira et al., 2011 [27]	Systematic review and meta-analysis	n=2,751 subjects. Adults undergoing various surgical procedures	Low dose of dexamethasone: less than 0.1 mg/kg; intermediate dose: 0.11-0.2 mg/kg; high dose: ≥0.21 mg/kg Timing: Preoperative or intraoperative. Control: Placebo or no treatment	Dexamethasone reduces postoperative pain at rest and with movement: At rest: ⩽4 h, −0.32 [0.47 to −0.18]; 24 h, −0.49 [−0.67 to −0.31]. With movement: ⩽ 4 h, −0.64 [−0.86 to −0.41]; 24 h, −0.47 [−0.71 to −0.24]. Opioid consumption is decreased with moderate and high doses: Moderate: −0.82 (−1.30 to −0.42); High: −0.85 (−1.24 to −0.46). Preoperative administration produces more consistent analgesic effects. Intermediate dose (0.11-0.2 mg/kg) is safe and effective for reducing postoperative pain and opioid consumption.
Laconi et al., 2024 [28]	Systematic review and meta-analysis	Non-obese adult population n=3943 patients	Intervention group: Perioperative administration of high dose of dexamethasone (≥0.2 mg/kg or ≥ 15 mg), or a corresponding dose of a systemic glucocorticoid. Control group: Placebo (placebo drug) or no treatment.	Mean difference (MD) in VAS scores at rest: 24 h: −6.18 mm 95% CI (−8.53, −3.83). Mean difference (MD) in VAS scores at motion: 24 h: −8.86 mm 95% CI (−11.82, −5.89). Reduction in opioid analgesic requirements: −10.00 mg; 95% CI (−13.65, −6.34). Odds ratio for PONV events: 0.29; 95% CI (0.24, 0.36). Odds ratio for major adverse events: 0.88; 95% CI (0.65, 1.19). Odds ratio for minor adverse events: 1.29; 95% CI (0.86, 1.92)

Taken together, the evidence supports the preoperative or intraoperative administration of intermediate-to-high dose dexamethasone as an effective strategy to enhance postoperative analgesia, reduce opioid consumption, and improve overall recovery outcomes (Table 1). While the meta-analysis by De Oliveira et al. (2011) [27] and more recent studies report no significant increase in major adverse events such as wound infections, clinicians should remain vigilant for potential risks, including transient hyperglycemia, particularly in diabetic patients, and theoretical infection concerns in immunocompromised individuals. These risks appear dose-dependent and should be weighed against the benefits of reduced opioid use and PONV. In populations with diabetes or immunosuppression, individualized risk assessment and perioperative glucose monitoring are recommended.

Overall synthesis and clinical implications

The collective evidence synthesized in this review supports a strong conclusion: non-opioid MMA has evolved from a theoretical strategy into a mature, evidence-based framework for perioperative care. While its adoption as a standard is increasingly widespread, the quality and consistency of evidence vary across surgical procedures and specific agent combinations. Nonetheless, consistent positive findings across a wide surgical spectrum, including outpatient cosmetic procedures [8,22], major abdominal surgery [14], and complex spine operations [10], demonstrate that these regimens provide postoperative analgesia that is at least non-inferior, and frequently superior, to traditional opioid-centric approaches. Notably, this superiority often arises not from large differences in pain scores but from achieving comparable analgesia while reducing opioid-related adverse effects, enhancing patient satisfaction, and accelerating key recovery metrics such as time to PACU discharge [21,22] and restoration of gastrointestinal function [18,19]. Implementation is most effective when tailored to specific surgical contexts, with emerging evidence supporting procedure-specific pairings such as dexamethasone and regional anesthesia for major abdominal surgery, scheduled acetaminophen and NSAIDs for orthopedic procedures, and gabapentinoids for spine surgery, among others.

Regional anesthesia and glucocorticoids represent two pivotal and complementary pillars of non-opioid MMA. Regional techniques, including fascial plane blocks, provide targeted analgesia that extends well beyond the immediate postoperative period. A 2024 meta-analysis by Pepper et al. found that regional anesthesia significantly reduces the risk of prolonged opioid use and chronic postsurgical pain for up to six months [25], positioning it not only as an acute pain intervention but also as a preventive strategy against pain chronification and long-term opioid dependence. Similarly, the role of glucocorticoids, particularly dexamethasone, has expanded considerably. Beyond their established antiemetic effects, systematic reviews show that intermediate-to-high dosing yields substantial reductions in resting and movement-associated pain and markedly decreases 24-hour opioid consumption [27,28]. This combined analgesic and antiemetic profile makes dexamethasone a uniquely efficient component of multimodal care.

The effectiveness of non-opioid MMA fundamentally arises from pharmacologic synergy and mechanistic diversity. No single non-opioid agent can fully substitute for a potent mu-opioid receptor agonist; however, strategic combinations act concurrently on multiple pain pathways, addressing peripheral inflammation (NSAIDs, glucocorticoids), central sensitization (NMDA antagonists, gabapentinoids), descending inhibitory modulation (α2-agonists, acetaminophen), and neuronal signal propagation (lidocaine, regional blocks). This mechanistic breadth explains the profound opioid-sparing effects observed in integrated regimens, such as the paracetamol-nefopam-ketoprofen (PNK) protocol [12] and the comprehensive intraoperative regimen described by Feld et al. [14], where opioid reduction frequently exceeds 80-90% [15].

A significant conceptual shift reflected in contemporary evidence is the movement from a narrow focus on pain intensity toward a holistic emphasis on recovery quality. The benefits of MMA extend far beyond numerical pain scales, yielding tangible improvements in clinically meaningful outcomes: earlier readiness for discharge [22], faster return of bowel function [18,19], reduced incidence of sedation [14] and postoperative nausea and vomiting [6,21,28], and consistently higher patient satisfaction [14,22] and adherence to analgesic protocols [22].

Nevertheless, implementation challenges remain, principally due to variability in study designs and the absence of a single, universally applicable regimen. Optimal MMA protocols are inherently procedure-specific and must account for individual patient characteristics. NSAID-based regimens may be ideal in ambulatory surgery settings for their efficiency and minimal impact on recovery time [22], whereas major abdominal operations benefit greatly from IV lidocaine and ketamine due to their synergistic opioid-sparing and prokinetic properties [14,18]. Surgeries with substantial somatic or incisional pain, including thoracic and breast procedures, derive marked benefit from regional anesthesia, while glucocorticoids provide broad advantages across nearly all operative contexts. Even in spine surgery, historically considered high-risk for NSAID use, evidence supports their safe and effective integration [10]. Thus, successful adoption requires thoughtful clinical judgment and institution-specific protocol development rather than rigid adherence to a single predefined regimen.

Limitations of the current evidence base

Despite strong support for the efficacy of non-opioid MMA, several limitations within the existing literature warrant attention. A primary challenge is the considerable variability in intervention components across studies. Differences in dosing strategies, timing of administration (preemptive vs postoperative), duration of therapy, and combinations of agents contribute to significant heterogeneity, complicating the comparison of outcomes and limiting opportunities for meta-analysis. Given this heterogeneity, caution is advised when directly comparing effect sizes across studies, as differences may reflect variations in protocol rather than true efficacy. This heterogeneity also makes it difficult to identify a universally optimal protocol for any given surgical procedure.

It is also important to acknowledge the methodological limitations inherent to this narrative review. As a narrative synthesis, this review did not follow a registered protocol or formal guidelines for systematic reviews (e.g., PRISMA), nor did it include a formal quality appraisal or risk-of-bias assessment of the included studies. While this approach allows for a broad, clinically oriented synthesis of a heterogeneous literature, it may affect the reproducibility and systematic rigor of our conclusions. Nevertheless, we prioritized higher-level evidence, such as RCTs, systematic reviews, and meta-analyses, in our interpretation and have been transparent about the sources and consistency of the evidence presented.

Generalizability is another concern. Many included studies are single-center trials, which, although internally robust, may not reflect the full spectrum of clinical settings characterized by diverse patient populations, perioperative workflows, and resource availability. Publication bias further complicates interpretation, as studies demonstrating neutral or negative findings may be underrepresented in the literature, potentially inflating perceived effect sizes.

Finally, an important evidence gap persists regarding long-term outcomes. Current data strongly support the short-term efficacy of MMA for acute postoperative pain, yet relatively few studies evaluate its impact on chronic postsurgical pain or new persistent opioid use. High-quality, long-term follow-up studies are needed to determine whether the short-term benefits of MMA translate into sustained reductions in opioid dependence and chronic pain syndromes.

A framework for implementation and future directions

To effectively translate the robust evidence supporting non-opioid MMA into routine clinical practice, a structured and scalable implementation framework is essential. First, healthcare institutions should prioritize procedure-specific protocol development. Creating standardized order sets for commonly performed surgeries, such as colorectal, orthopedic, and gynecologic procedures, can streamline clinical decision-making, promote the use of synergistic 2-4 agent combinations, and reduce reliance on variable discretionary prescribing.

Second, successful MMA regimens emphasize preemptive and scheduled dosing. Studies such as those by Beloeil et al. (2019 [12]) and Nguyen et al. (2018 [21]) demonstrate that maintaining consistent therapeutic levels of non-opioid agents is more effective in preventing pain than treating established pain after onset. Routine scheduling of medications, rather than PRN administration, should therefore be standard practice.

Third, proactive patient education is essential. Patients should be counseled that the goal is functional recovery and comfort with minimal opioid exposure, and not complete elimination of pain. Clear expectations can improve adherence, reduce anxiety, and enhance satisfaction with the analgesic plan.

Looking ahead, future research must focus on addressing current evidence gaps and refining clinical pathways. Key priorities include head-to-head comparisons of different multimodal bundles to identify the most effective and efficient combinations for distinct procedures; cost-effectiveness analyses to support institutional adoption; and dedicated studies examining the safety and efficacy of MMA regimens in vulnerable populations such as older adults or individuals with substance use disorders. Finally, long-term follow-up studies are crucial to evaluate whether MMA protocols reduce chronic postsurgical pain and prevent persistent opioid use, thereby advancing both perioperative care quality and long-term public health outcomes.

## Conclusions

This review establishes non-opioid MMA as an evidence-based standard for postoperative pain management. Synergistic combinations of NSAIDs, acetaminophen, NMDA antagonists, lidocaine, α2-agonists, regional anesthesia techniques, and glucocorticoids consistently provide effective analgesia while substantially reducing opioid requirements and opioid-related adverse effects. The incorporation of regional anesthesia offers the additional benefit of reducing long-term risks such as chronic postsurgical pain and prolonged opioid use, whereas glucocorticoids simultaneously and efficiently address both pain and postoperative nausea. Together, these modalities form a comprehensive strategy that aligns closely with ERAS principles and improves patient recovery, satisfaction, and safety.

Going forward, clinical implementation should prioritize the development and dissemination of procedure-specific protocols that integrate these complementary modalities. Future research is needed to optimize drug and technique combinations, evaluate long-term outcomes, and identify and overcome barriers to implementation across diverse clinical settings. Broader adoption of these opioid-sparing strategies represents a critical advancement toward safer surgical care and a practical, multifaceted response to the ongoing opioid crisis.
